# Progressive disease in glioblastoma: Benefits and limitations of semi-automated volumetry

**DOI:** 10.1371/journal.pone.0173112

**Published:** 2017-02-28

**Authors:** Thomas Huber, Georgina Alber, Stefanie Bette, Johannes Kaesmacher, Tobias Boeckh-Behrens, Jens Gempt, Florian Ringel, Hanno M. Specht, Bernhard Meyer, Claus Zimmer, Benedikt Wiestler, Jan S. Kirschke

**Affiliations:** 1 Department of Neuroradiology, Klinikum rechts der Isar, Technical University of Munich, Germany; 2 Institute for Clinical Radiology, Ludwig-Maximilians-University Hospital, Munich, Germany; 3 Department of Neurosurgery, Klinikum rechts der Isar, Technical University of Munich, Germany; 4 Department of Radiation Oncology, Klinikum rechts der Isar, Technical University of Munich, Germany; German Cancer Research Center (DKFZ), GERMANY

## Abstract

**Purpose:**

Unambiguous evaluation of glioblastoma (GB) progression is crucial, both for clinical trials as well as day by day routine management of GB patients. 3D-volumetry in the follow-up of GB provides quantitative data on tumor extent and growth, and therefore has the potential to facilitate objective disease assessment. The present study investigated the utility of absolute changes in volume (delta) or regional, segmentation-based subtractions for detecting disease progression in longitudinal MRI follow-ups.

**Methods:**

165 high resolution 3-Tesla MRIs of 30 GB patients (23m, mean age 60.2y) were retrospectively included in this single center study. Contrast enhancement (CV) and tumor-related signal alterations in FLAIR images (FV) were semi-automatically segmented. Delta volume (dCV, dFV) and regional subtractions (sCV, sFV) were calculated. Disease progression was classified for every follow-up according to histopathologic results, decisions of the local multidisciplinary CNS tumor board and a consensus rating of the neuro-radiologic report.

**Results:**

A generalized logistic mixed model for disease progression (yes / no) with dCV, dFV, sCV and sFV as input variables revealed that only dCV was significantly associated with prediction of disease progression (P = .005). Delta volume had a better accuracy than regional, segmentation-based subtractions (79% versus 72%) and a higher area under the curve by trend in ROC curves (.83 versus .75).

**Conclusion:**

Absolute volume changes of the contrast enhancing tumor part were the most accurate volumetric determinant to detect progressive disease in assessment of GB and outweighed FLAIR changes as well as regional, segmentation-based image subtractions. This parameter might be useful in upcoming objective response criteria for glioblastoma.

## Introduction

MR imaging plays a central role in response assessment of glioblastoma (GB), both in clinical trials as well as in the daily clinical management of GB patients. To avoid inter-observer bias of visual image interpretation, means to provide a more objective imaging assessment by quantifying image information like GB growth are receiving increasing attention [1–4]. Many efforts have been made to reliably quantify glioblastomas in MR images [2–18]. Response evaluation criteria in solid tumors (RECIST), Macdonald criteria or response assessment in neuro-oncology working group (RANO) criteria apply one- or two-dimensional assessments such as longest tumor diameter or the products of perpendicular diameters respectively [2–4]. Even though RANO criteria are broadly and easily applicable and help to standardize response evaluation, reproducibility is low and there are several limitations, especially for irregular-shaped tumors or masses around resection cavities or cysts [4,19].

Three-dimensional volumetric tumor assessment using MR image segmentations might overcome current limitations of uni- and biplanar assessments and offers an elegant way to quantify image information [5,6,8,18]. There is a strong need and a high interest in reliable image segmentation techniques, which gets mirrored in the exponential rise of studies on image segmentation [5]. In general, three different segmentation techniques are widely applied: manual, semi-automated and fully automated segmentation techniques. Manual segmentations are still regarded as the gold standard in many imaging studies but require a human rater which makes them often time consuming and prone to bias [18]. Semi-automated segmentation tools often apply intelligent region-growing algorithms that assist the rater during the delineation, saving time and increasing homogeneity of segmentations [13–15,18,20,21]. Automated segmentation techniques offer constant results but still have several limitations in terms of precision or unexpected signal alterations like in postoperative MRI of GB [5,6,16,17,22].

Contrast enhanced T1-weighted and T2-weighted fluid-attenuated-inversion-recovery (FLAIR) sequences are crucial for glioma evaluation and are consequently part of a consensus recommendation for respective imaging protocols [23]. With modern segmentation techniques being widely-used, quantification of tumor volume is now feasible in MRI follow-ups with only moderate effort. Even though reliable segmentation techniques are progressively studied and volumetric assessments are increasingly applied in new imaging studies [6–8], their diagnostic benefit still remains uncertain [5,11,12]. Further it is not clear which volumetric determinant should be addressed in the follow-up of GB since many studies did either not include tumor-related FLAIR signal changes or regional, segmentation-based subtractions in GB [6–8].

The aim of this study was to analyze if semi-automated volumetric assessments in GB can predict disease progression in routine MRI follow-ups. Therefore, the change of absolute tumor volumes (delta) and regional volume changes revealed by image subtractions were studied and compared to an expert consensus decision based on the best available data for each follow-up, including histopathological results, decisions of the local multidisciplinary CNS tumor board and neuro-radiologic reports.

## Methods

### Subjects

30 consecutive GB patients (23m, mean age 60.2y) of the local neuro-oncology clinic with at least three high-resolution MRI follow-ups between 10/2013 and 03/2015 were included in this single-center retrospective study. Altogether, 330 longitudinal tumor segmentations were performed on high resolution 3D sequences as specified below. Diagnosis of GB was pathologically confirmed in all cases at the local Department of Neuropathology. Patient records were de-identified and analyzed anonymously.

### MR imaging

High-resolution MR imaging was done on either a 3 Tesla (T) Achieva scanner (Philips Medical Systems, The Netherlands) or a 3T Verio scanner (Siemens Healthcare, Erlangen, Germany) at the local Department of Neuroradiology. 3D-spoiled gradient echo sequences were applied either with an 8-channel or 16-channel phased array head coil. T2-weighted FLAIR sequences were acquired with a spatial resolution of 1.04 x 1.04 x 1.12 mm (TR/TE of 4800/278, 3T Achieva) and 1 x 1 x 1 mm (TR/TE of 5000/395 ms, 3T Verio). T1-weighted magnetization prepared rapid gradient echo (MPRage) sequences were acquired with a spatial resolution of 1 x 1 x 1 mm (TR/TE 9/4 ms, 3T Achieva) and 1.1 x 1.1 x 1 mm (TR/TE 1900/2.45 ms, 3T Verio). The contrast mediums Magnograf^®^ (MaRoTrast, Jena, Germany) or Dotarem^®^ (Guerbet, Villepinte, France) were administered with an automated injection system (Spectris Solaris EP, Siemens Medical, Erlangen, Germany).

### Tumor segmentation

**Fig 1:**
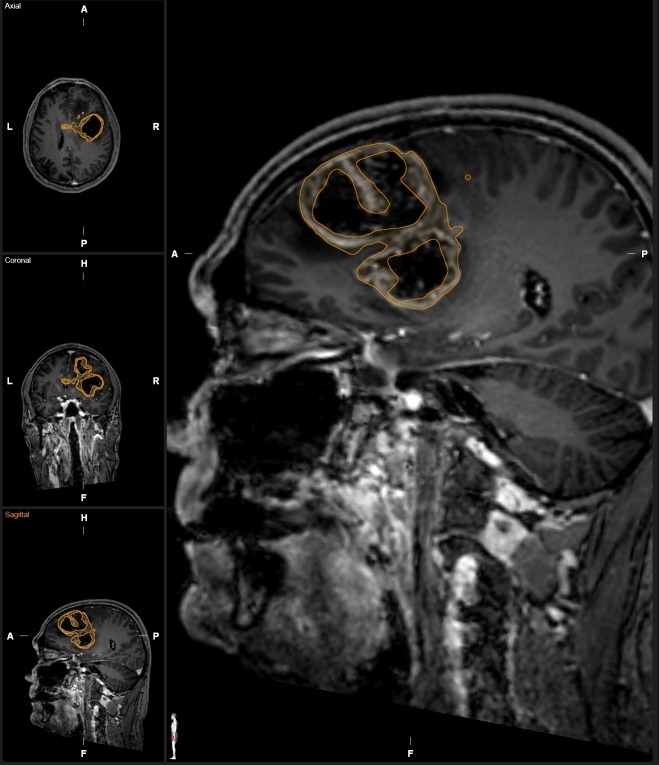
Example segmentation of a complex GB. Contrast enhancing tumor volume was delineated on a contrast enhanced MPRage image set rendering a complex tumor shape. Necrotic areas were avoided during segmentation as it was feasible. The small spot located posterior to the segmentation on the magnified sagittal image represents the “brush” of the semi-automated region growing tool.

All tumor segmentations were done semi-automatically with ‘Smartbrush’, a tool of Brainlab Elements (Brainlab, Feldkirchen, Germany). Smartbrush is a software solution for image segmentation, based on a region-growing algorithm that can be reliably used for tumor segmentations in GB [18]. Therefore a 2D-segmentation was drawn in an axial image and another 2D-segmentation was delineated in a coronal image. These two segmentations were used to automatically generate a rough 3D-interpolation of the tumor. The 3D-interpolation was then manually corrected by adding or erasing certain areas. 165 segmentations of FLAIR images were performed to measure the volume of FLAIR-hyperintense, tumor related changes (FV). The corresponding 165 MPRage images were segmented in parallel to assess contrast enhancing tumor parts (CV). Structures and signal intensities that were not tumor-related, e. g microangiopathy, ventricular plexus and vessels were not segmented. Further, resection cavities, unspecific postoperative enhancement and tumor cysts were not included in the segmentation (Fig 1) [24]. To exclude blood and postoperative blood residuals, a native MPRage sequence and the corresponding subtraction between the contrast-enhanced MPRage and the native MPRage sequence was displayed on a separate screen of the segmentation workstation.

### Regional, segmentation-based subtractions and change of absolute tumor volumes

**Fig 2:**
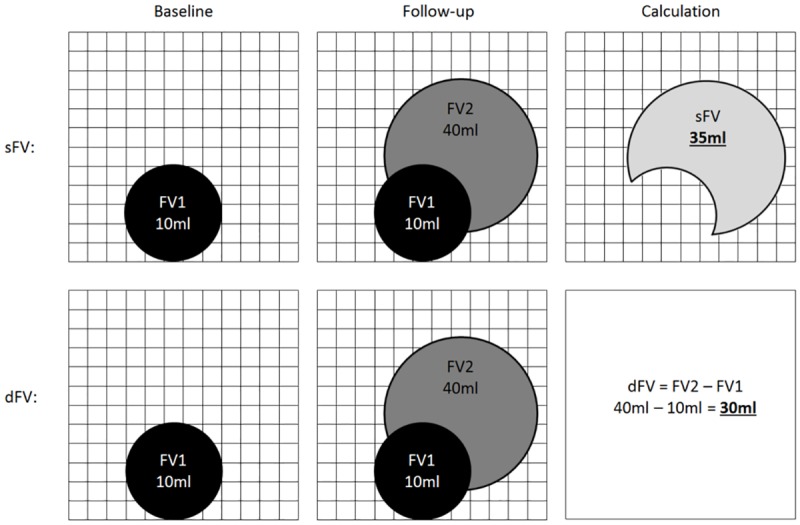
Scheme for calculations of regional, segmentation-based subtractions (sFV, upper row) and absolute changes of tumor volume (dFV, lower row). The FLAIR segmentation of the baseline MRI is referred to as FV1, the segmentation of the follow-up MRI as FV2. For regional, segmentation-based subtractions (sFV) a voxel-wise subtraction was performed. Only overlapping voxels of FV1 and FV2 were subtracted. For the absolute change of tumor volume the total change of volume between FV2 and FV1 was calculated by subtraction.

We used two different methods of determining the quantifiable change between tumor volume in two consecutive MRI scans, the regional, segmentation-based approach and the change of absolute tumor volume. Both methods are schematically illustrated in Fig 2.

**Fig 3:**
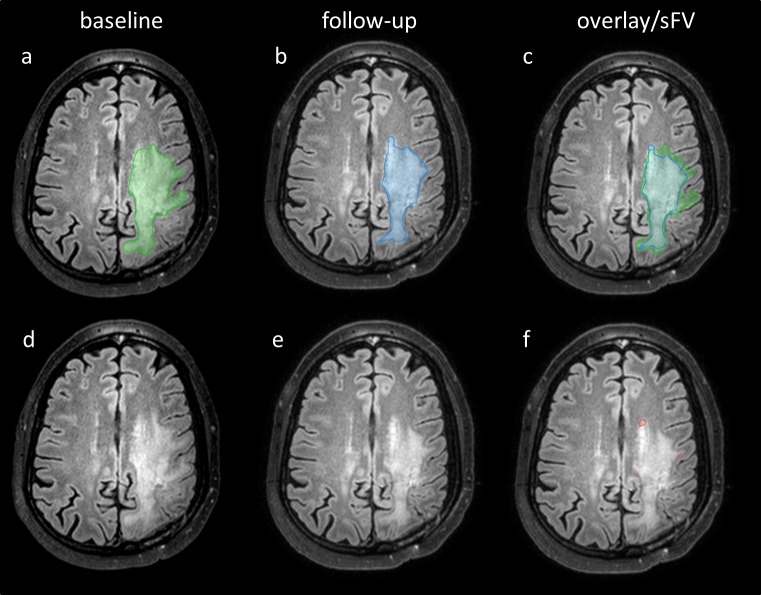
Examples of FLAIR segmentations and regional, segmentation-based subtractions. Axial FLAIR slices of the baseline MRI (a, d) and the follow-up MRI (b, c, e, f) of a patient with GB are shown. Segmentation of baseline FV (a, green), follow-up FV (b, blue) and an overlay image of both segmentations (c) is presented. Baseline FLAIR image (d) and follow-up FLAIR image (e) are displayed without segmentations. The regional, segmentation-based subtraction of FV (sFV) is also shown (f, red).

**Fig 4:**
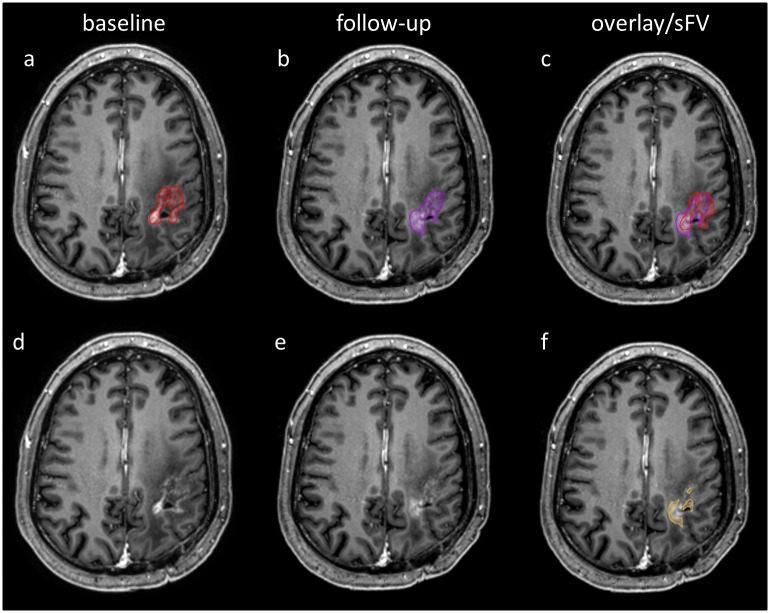
Examples of contrast-enhanced MPRage segmentations and regional, segmentation-based subtractions. Axial MPRage slices of the baseline MRI (a, d) and the follow-up MRI (b, c, e, f) of a patient with GB are shown. Segmentation of baseline CV (a, red), follow-up CV (b, purple) and an overlay image of both segmentations (c) is presented. Baseline MPRage image (d) and follow-up MPRage image (e) are displayed without segmentations. The regional, segmentation-based subtraction of CV (sCV) is also shown (f, yellow).

For regional, segmentation-based subtractions we registered MR images and tumor segmentations (CV, FV) applying the built-in rigid image fusion tool of Brainlab based on trust region methods [25]. Success of image registration was carefully reviewed, corrected if necessary and exported to iPlan 3.0 (Brainlab, Feldkirchen, Germany). Segmentation-based subtractions of CV and FV between two consecutive, registered MRI follow-ups were performed with the logical image operation tool of iPlan 3.0 and are referred to as sCV and sFV. Therefore a voxel-wise subtraction between the tumor segmentation of the follow-up MRI and the baseline MRI was automatically performed by the software. Regional, segmentation based subtractions have the advantage of still maintaining information about the voxel localization since both images are registered (Figs 3 and 4). We further calculated the absolute change of tumor volume between two consecutive MRI scans. Therefore we subtracted the absolute tumor volume of the preceding MRI of the absolute tumor volume of the current MRI.

### Correction for precision error

All segmentations were done by one single rater with neuro-radiologic expertise (GA) to exclude inter-rater bias [18]. As previously described, the minimal change in volume that can be considered ‘significant’ (least significant change, LSC) for single rater segmentations between two consecutive segmentations was previously reported as 35.2% for CV segmentations and 14.4% for FLAIR segmentations using this semi-automated method [18]. We applied a transformation and divided all delta volumes by the respective absolute LSC to get relative ‘corrected’ delta volumes. This approach was chosen since it led to a scaling of tumor volumes making them better comparable. The corrected delta volumes are termed dCV for contrast enhancing lesions and dFV for tumor-related FLAIR changes.

### Radiologic Consensus (RC)

Neuro-radiologic reports were reviewed independently by two experienced neuroradiologists (TH, 3 years of experience; JK, 2 years of experience) and rated on a nominal scale as follows: 0 = initial diagnosis; 1 = immediate postoperative MRI; 2 = disease regression; 3 = stable disease; 4 = uncertain disease progression (e.g. possible pseudo-progression following radiotherapy); 5 = disease progression. Inter-rater reliability between the two raters was assessed by Cohen’s kappa coefficient (κ) and can range between 0 (random agreement) and 1 (perfect agreement) [26]. κ > 0.8 is termed ‘almost perfect agreement’ in this study [27]. Discrepant ratings were reviewed in a separate session and evaluated in consensus (RC).

### Multidisciplinary Consensus (MC)

For each follow-up, additional data was obtained from the clinical information system, including histopathologic results from biopsies/resections and the decisions of the local multidisciplinary central nervous system (CNS) tumor board. The CNS tumor board is a weekly meeting of neuro-oncology specialists in the field of neurology, neurosurgery, neuroradiology, neuropathology, radiation oncology and nuclear medicine at our institution. Board decisions are based on current state of the art techniques in neuro-oncology, including assessments of RANO response criteria and results of additional positron emission tomography (PET) scans. Each follow-up was rated on the same nominal scale as RC to obtain the best possible multidisciplinary consensus. The following hierarchical steps were taken for rating of MC: Whenever histopathologic results were available for the follow-up (16.4%) MC was accordingly rated. If no histopathologic results were available (83.6%), MC was rated based on the decision of the CNS tumor board (42%). For the remaining cases MC was rated similar to RC.

### Generalized Linear Mixed-effects Model (GLMM)

A GLMM using a logistic link function was calculated, with MC as dependent binary variable (progression yes / no) and delta volumes (dCV, dFV) and regional, segmentation-based subtractions (sCV, sFV) as input. In addition, patient number was included as a random effect in this model. To further compare delta volumes and regional subtractions, we generated separate logistic mixed-effects models for both. To account for model bias, we performed 5-fold cross-validation of both these models and DeLong’s test for correlated ROC curves using the R package “pROC” [28]. Only cross-validated performance measures (i.e. accuracy) are reported. We performed an exact binomial test, using the rate of progressions as per MC (i.e. the “no information rate”) as probability of success, to assess whether the classification accuracy was above chance.

### Statistical analysis

Statistical calculations in this study were done with IBM SPSS Statistics, release 23.0 (IBM, Armonk, NY, USA) and R version 3.3.1 (www.r-project.org). For illustrations Brainlab Elements (Brainlab, Feldkirchen, Germany), Power Point 2010 (Microsoft, Redmond, USA), R and Adobe Photoshop CS4 (Adobe Systems, CA, USA) were used. Asterisks indicate significant results: ‘**’ P < .01, ‘*’ P < .05.

## Results

Median CV was 2.80 ml and median FV was 50.10 ml, the low median CV in part owing to the effect that follow-ups immediately after tumor resections were included (Table 1). Agreement between the two raters of the neuro-radiologic reports showed almost perfect agreement (κ = .82, P < .001). Accordance between RC and MC was almost perfect as well (κ = .923, P < .001), underlining a high agreement between radiologic and later multidisciplinary consensus. RC and MC only differed in 9 follow-up evaluations. The study included 165 MR imaging time points, leading to 135 MR follow-ups. 26 immediate postoperative MR examinations were excluded from further GLMM analysis.

**Table 1:** Patient characteristics and technical parameters.

	Number (n), mean +/- SD or median with 25% and 75% percentile
patients (n)	30 (100%)
male / female (n)	23 / 7 (77% / 23%)
age, years (mean +/- SD)	60.2 +/- 14.99
MGMT status (n = 27)	
methylated	13 (48%)
unmethylated	14 (52%)
IDH status (n = 21)	
wildtype (n)	20 (95%)
mutation (n)	1 (5%)
Chemotherapy	
Temozolomide (n)	26 (87%)
Bevacizumab (n)	2 (7%)
Radiotherapy	
received (n)	30 (100%)
initial dose in Gy (n = 28)	60 (60–60)
MRI scans (n)	
3	6
4	3
5	7
6	7
7	4
8	1
10	1
12	1
CV segmentations (n)	165
CV in ml (median)	2.80 (0.30–10.90)
dCV in ml (median)	0.00 (-2.60–3.40)
sCV in ml (median)	1.49 (0.11–6.24)
FV segmentations (n)	165
FV in ml (median)	50.10 (26.20–92.40)
dFV in ml (median)	0.80 (-16.10–19.30)
sFV in ml (median)	17.22 (7.48–35.18)
RC—MC categories	
0 “initial diagnosis” (n)	6 (3.6%)–6 (3.6%)
1 “postoperative” (n)	33 (20.0%)–33 (20.0%)
2 “regression” (n)	11 (6.7%)–11 (6.7%)
3 “stable” (n)	19 (11.5%)–17 (10.3%)
4 “uncertain progression” (n)	21 (12.7%)–15 (9.1%)
5 “progression” (n)	75 (45.5%)–83 (50.3%)

SD = standard deviation, CV = contrast enhancing volume, dCV = absolute change of CV between two consecutive MRI time points, sCV = regional, segmentation-based subtractions of CV between two consecutive MRI time points, FV = FLAIR hyperintense volume, dFV = absolute change of FV between two consecutive MRI time points, sFV = regional, segmentation-based subtractions of FV between two consecutive MRI time points, RC = radiologic consensus, MC = multidisciplinary consensus.

**Fig 5:**
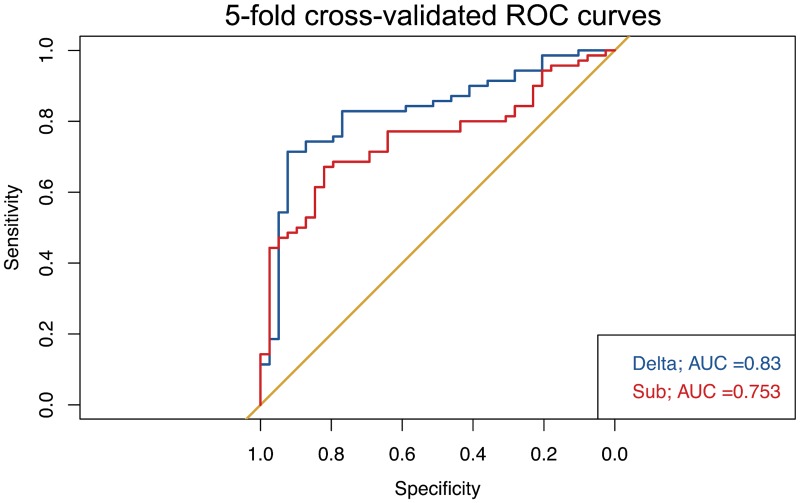
ROC-curves for volumetric determinants. Five-fold cross-validated (CV) ROC-curves for either changes in absolute volume (delta) or regional volume subtractions (sub) for the prediction of disease progression.

A GLMM was calculated, using MC dichotomized for ‘disease progression’ (yes / no) as dependent variable and the following input variables: dCV, dFV, sCV and sFV. In this model, “patient” was included as a random effect. Only dCV was significantly associated with prediction of disease progression (P = .005), whereas neither dFV nor the regional, segmentation-based subtractions showed significant effects (Table 2). To compare delta volumes and regional subtractions, we calculated separate GLMMs for both and computed the corresponding ROC curves after a 5-fold cross validation (Fig 5). The cross-validated area under the curve (AUC) of delta volumes was higher (.83) than the AUC for the volume subtractions (.75). However, DeLong’s test did not show a significant difference between the ROC curves (P = .162).

**Table 2:** Results of the GLMM for dichotomized MC (‘disease progression’: Yes / No) and the following input variables: DCV, dFV, sCV and sFV.

	Estimate	Std. Error	P
(Intercept)	-.782	.42488	.06569
dCV	.40383	.14461	.00523**
dFV	-.08750	.06032	.14689
sCV	.21685	.11967	.06999
sFV	.02769	.02171	.20227

dCV = absolute change of CV between two consecutive MRI time points, dFV = absolute change of FV between two consecutive MRI time points, sCV = regional, segmentation-based subtractions of CV between two consecutive MRI time points, sFV = regional, segmentation-based subtractions of FV between two consecutive MRI time points; Asterisks indicate significant results:

** P < .01

**Fig 6:**
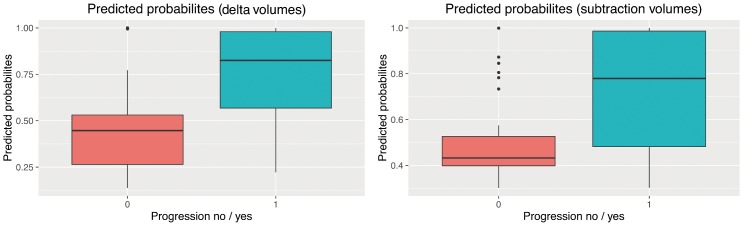
Prediction probabilities for disease progression. The prediction probabilities for the binary decision “disease progression” (yes / no) for changes in absolute volumes (left) and regional subtractions (right). Note that while the boxes in Fig 6 (left) do not overlap, this is the case in Fig 6 (right), indicating that a model based on delta volumes better separates progressive from non-progressive follow-up examinations.

The GLMM for delta volumes (dCV, dFV) tended to have a higher diagnostic accuracy (correctly classified ‘disease progression’) than the GLMM for regional, segmentation-based subtractions (AUC: .789 [95% CI: .700–.861] versus AUC .725 [95% CI: .631–.806]). For both models, this accuracy was above chance (assuming a no information rate of .642), although just barely (P = .0427) in the case of the GLMM based on regional subtractions. Predicted probabilities for disease progression showed a clearer separation when using delta volumes compared to regional subtractions (Fig 6). However, accuracy for RC was .973 [95% CI: .922–.994] and hence well above the volumetric parameters.

## Discussion

Three main conclusions may be drawn from our study: (i) The absolute change of contrast enhancing tumor volume is the most important volumetric determinant to detect progressive disease. (ii) Changes in absolute tumor volume outweigh changes in regional, segmentation-based subtractions by trend. (iii) Conventional visual evaluations of MRI images by experts are yet more accurate than segmentation-based volumetric assessments.

Our results provide evidence that absolute volumetric changes in contrast enhancing GB lesions between two consecutive MRI follow-ups are the main imaging determinant in tumor volumetry for the detection of progressive disease. To minimize technical bias, we only included 3D high-resolution imaging (both for T1 and FLAIR) and all segmentations were done by a single rater which excludes inter-rater bias [18,29]. Further, all data were corrected for the precision error arising from intra-rater variability [18,30,31]. Even though the precision error for segmentations of contrast enhancement was previously reported to be more than two times higher than for tumor-related FLAIR changes [18], the corrected change in contrast enhancement still outweighed FLAIR changes for the prediction of progressive disease.

The current RANO criteria only suggest two-dimensional quantification of contrast enhancing lesions, whereas FLAIR images are only qualitatively rated in RANO [2–4]. This approach is supported by our study. Certain limitations for the assessment of contrast enhancement apply for RANO criteria, i.e. non-measurable lesions < 10 mm in diameter or tumors around resection cavities and cysts, which cannot adequately be described by a perpendicular diameter. Our generalized volumetric approach was applied at every MRI follow-up and even small or irregular-shaped lesions could be reliably assessed. However, for the GLMM we excluded patients with immediate post-operative MRI. Our rationale for the exclusion was that a) postoperative signal alterations were difficult to distinguish from tumor during segmentation and b) the unusual large decrease of tumor volume in immediate post-operative MRI might have led to an overestimation of the GLMM resulting in an overly selective model which weakens the validity for routine MRI follow-ups.

Contrast enhancement is still the best surrogate for proliferating tumor cells, however it only reflects a disrupted blood-brain-barrier (BBB) and GB usually extends well beyond the contrast enhancing margins [32,33]. Other causes for a disturbed BBB must be considered when contrast enhancement is assessed, as radiotherapy, (perioperative) ischemia or novel anti-angiogenic dugs like bevacizumab affect the BBB and subsequent MRI follow-ups [34,35]. Since we evaluated routine MRI follow-ups, the vast majority of patients have received prior adjuvant chemo-/radiotherapies. Even though segmentations were performed with great care, therapy-associated changes of contrast enhancement might be contained in the segmented volumes. Notwithstanding this limitation, our approach showed a fair diagnostic accuracy and can be applied at any MRI follow-up.

Progressive FLAIR changes are only qualitatively included in the current RANO criteria [4]. As opposed to contrast enhancement, RANO only requires a “significant” increase in FLAIR signal to establish disease progression. Further complicating FLAIR assessment beyond this subjective criterion, several competing causes for FLAIR signal increase like radiation therapy, ischemia, seizures, decreasing doses of corticosteroids, postoperative changes or other treatment effects must be taken in consideration when evaluating FLAIR images [4, 36]. Even though previous studies suggested cut-offs for progressive FLAIR changes [37], our study did not establish a significant association of changes in FLAIR signal with prediction of disease progression. As we performed volumetric assessments in every consecutive MRI, the above mentioned exceptions for FLAIR progression in RANO apply as limitations to our approach. However, our method aimed to simplify quantitative assessments in routine MRI follow-ups without having too many exceptions or non-measurable cases.

Regional, segmentation-based volume subtractions performed on registered MR images did not provide an advantage compared to absolute changes in volume. There are two main explanations that should be considered. First, local inaccuracies during semi-automated segmentations might account for regional subtraction errors, even though segmentations were done with great care. Second, regional volume subtractions largely depend on the quality of image registration, which is an important and well-studied field in imaging [25,38–41]. However image registration has certain limitations when applied to postoperative MRI since the resection cavity, brain shift or signal changes due to edema, ischemia or bleeding complicate its applicability. Rigid registration techniques are prone to misregistration at the resection margins in postoperative MRI. Even though we excluded immediate postoperative MRI, (small) registration errors might explain the weak performance of regional subtractions in this study since progressive disease usually occurs at the resection margins. Ideally non-rigid image registration techniques should be applied and further developed [38,40]. However, these techniques sometimes apply spatial transformations of MR images which could lead to inaccurate tumor volumes [41]. Despite these limitations, human raters might still benefit from regional subtractions since local changes can be easily visualized and evaluated.

Previous authors reported good correlations between established one- or two-dimensional assessments and novel three-dimensional volumetry in GB raising the question of its additional value [9–12]. Semi-automated or manual segmentations require more time and the development and implementation of appropriate software solutions, which can limit the usability and availability in routine clinical evaluation of follow-up MRIs. Nevertheless, automated segmentation techniques are being developed which can save time and exclude human bias [5,16]. Further, it is likely that automated segmentations might get integrated in the picture archiving and communication system (PACS), making them more available. In the future, volumetric assessments might be part of novel objective response criteria since their use in current clinical trials is already increasing [6–8]. However, sole volumetric assessments are still outperformed by experienced human raters. Current volumetric assessments in GB might therefore primarily assist professional human raters in routine MR follow-up examinations. Assessing an additional diagnostic benefit would require very large cohorts since agreement between conventional radiologic consensus and multidisciplinary consensus was high in our study.

Besides the usual limitations of a retrospective study design some further restrictions should be considered when interpreting the results in this study. The dependent binary variable MC in the GLMM was evaluated to the best of our knowledge, taking histologic results and consensus decisions of the CNS tumor board based on RANO criteria into account. Though, in almost half of all cases the radiologic consensus decision was the only available variable to rate MC. Second, we intended to quantify tumor volumes in all follow-ups. Therefore, therapy-related changes due to adjuvant radio- and chemotherapies are present in some MR images and could not be distinguished from tumor volumes during segmentations in a few cases. Third, even small contrast enhancing lesions were included in the segmentations since 3D-high-resolution MRI data was used. However, in some cases minor signal alterations, i.e. enhancement at the rim of the resection cavity, could not be reliably distinguished from postoperative scaring or unspecific enhancement. In particular, small segmentations were prone to wrong registration in image fusion and might lead to higher results in image subtractions due to the absence of overlapping regions.

## Conclusion

In longitudinal MRI follow-ups of glioblastoma, the change in absolute volume of contrast enhancement is the most important volumetric parameter to detect progressive disease and is an objective imaging determinant. Regional, segmentation-based image subtractions are less reliable by trend, probably because of local inaccuracies of current image segmentation and registration techniques. Conventional visual evaluation of imaging experts is yet more accurate than volumetric assessments in MRI follow-ups of glioblastoma.

### Ethical standards

This study was approved by the local ethics committee at the Klinikum rechts der Isar of the Technical University of Munich, Germany, in accordance with the ethical standards of the 1964 Declaration of Helsinki and its later amendments [42].

## Supporting information

S1 Information Volumetric dataset. (XLSX)
